# Whole brain volume changes and its correlation with clinical symptom severity in patients with schizophrenia: A DARTEL-based VBM study

**DOI:** 10.1371/journal.pone.0177251

**Published:** 2017-05-17

**Authors:** Gwang-Won Kim, Yun-Hyeon Kim, Gwang-Woo Jeong

**Affiliations:** 1 Research Institute for Medical Imaging, Chonnam National University Medical School, Gwangju, Republic of Korea; 2 Department of Radiology, Chonnam National University Medical School, Chonnam National University Hospital, Gwangju, Republic of Korea; Chiba Daigaku, JAPAN

## Abstract

The purpose of this study was to evaluate gray matter (GM) and white matter (WM) volume alterations in whole-brain structures in patients with schizophrenia and healthy controls using voxel-based morphometry (VBM), and further to assess the correlation between GM and WM volume variations and symptom severity in schizophrenia. A total of 22 patients with schizophrenia and 22 age-matched healthy controls participated. Magnetic resonance image data were processed using SPM8 software with diffeomorphic anatomical registration via an exponentiated Lie algebra (DARTEL) algorithm. Patients with schizophrenia exhibited significantly decreased GM volumes of the insula, superior temporal gyrus (STG), gyrus rectus, and anterior cingulate cortex (ACC) compared with healthy controls. The GM volumes of the STG and gyrus rectus were negatively correlated with the positive scales on the Positive and Negative Syndrome Scale (PANSS) and those of the STG and ACC were negatively correlated with the negative scales. The durations of illness in schizophrenia were negatively correlated with the GM volumes of the insula, STG, and ACC. Patients with schizophrenia exhibited significantly decreased WM volumes of the superior frontal gyrus, inferior temporal gyrus, and STG. The WM volumes of the STG were negatively correlated with the duration of illness. Our findings suggest that GM and WM volume abnormalities in the STG are associated with the psychopathology of schizophrenia.

## Introduction

Schizophrenia is a severe mental illness characterized by deficits in cognition and emotion [1]. Brain structural abnormalities are detectable in most individuals afflicted with schizophrenia during the chronic stages [2]. A 3% decrease in whole-brain volume has been observed in patients with schizophrenia, and this decrease is more prominent in gray matter (GM; 2%) than in white matter (WM; 1%) [3,4]. Volumetric GM abnormalities have become more important than ever in understanding the neuropathology of schizophrenia.

Early morphological studies [5,6] of schizophrenia primarily assessed specific brain regions of interest (ROIs). Since the 2000s, voxel-based morphometry (VBM) has grown in popularity regarding the study of psychiatric disorders and diseases. This automated voxel-based whole-brain analysis technique is able to evaluate overall GM and WM volume differences between groups using statistical tests across all voxels in a given image set [7–11]. The standard VBM introduced by Ashburner and Friston [8] and the optimized VBM proposed by Good et al. [10] have been used to detect the presence of neurological diseases. According to a meta-analysis of morphometric studies of schizophrenia, patients with this condition exhibit reduced GM volumes in the insula, anterior cingulate cortex (ACC), parahippocampal gyrus, middle frontal gyrus, postcentral gyrus, and thalamus [12]. Other VBM studies [13–15] have found GM deficits in brain structures such as the superior/medial temporal gyri, inferior/medial frontal gyri, inferior parietal lobe, insula, thalamus, and basal ganglia in patients with schizophrenia. However, the precise neural morphometric deviations associated with schizophrenic symptoms have not yet been specified; the discrepancies between the results of these studies are likely attributable to differences in magnetic resonance imaging (MRI) acquisition methodologies, the (pre)processing steps for VBM and statistical analysis.

Ashburner [9] developed the diffeomorphic anatomical registration through an exponentiated Lie algebra (DARTEL) algorithm to more accurately evaluate GM and WM volumes. DARTEL-based VBM is capable of providing more precise inter-subject alignment in association with image registration and segmentation compared with the standard VBM and optimized VBM techniques [7]. Asami et al. [16] reported that patients with first-episode schizophrenia showed GM volume reductions in widespread neocortical regions including the superior temporal gyrus (STG) and the frontal and parietal gyri, as well as the limbic regions using SPM5 software with the DARTEL algorithm. Another VBM study [17] using SPM8 software reported that patients with late- and early-onset schizophrenia exhibited decreased GM volumes in the insula, STG, and orbitofrontal gyrus. The Positive and Negative Syndrome Scale (PANSS) is the score for accessing the symptom severity of patients with schizophrenia, which is potentially an important factor for evaluating the etiology of schizophrenia. Therefore, a research associated with GM volume measurement in relation to the PANSS is important for patients with schizophrenia. However, no DARTEL-based VBM study has assessed the relationship between GM and WM volume alterations and specific subscales of the PANSS in patients with chronic schizophrenia. We hypothesized that GM and WM volume variations are associated with the specific PANSS subscales.

Sex differences in schizophrenia are one of the most consistently reported aspects of the disease [18]. The incidence of schizophrenia in men is approximately 1.5 times higher than that in women [19]. Several MRI studies [20,21] have focused on assessing the brain volume changes associated with the sex differences in schizophrenia. Nopoulos et al. [20] found that male and female patients with schizophrenia show the same pattern of structural brain abnormalities, but male patients appear to manifest more severe symptoms. However, Goldstein et al. [21] found that female patients show a larger total cortical volume, but a smaller total cerebral volume compared with male patients. DARTEL-based VBM of whole-brain structures may advantageously provide more valuable information regarding the gender-dependent brain abnormality observed in patients with schizophrenia.

Thus, the primary purpose of this study was to compare GM and WM volume alterations over whole-brain areas between healthy controls and patients with schizophrenia using DARTEL-based VBM, and further to assess the correlation between GM and WM volume variations and symptom severity in patients with schizophrenia. In addition, we evaluated the gender-specific brain abnormalities associated with the GM and WM volumes of patients with schizophrenia.

## Materials and methods

### Ethics

This study was approved by the Institutional Review Board of Chonbuk National University Hospital. All volunteers received an explanation of the experimental procedure before MR scanning and provided written informed consent. The capacity of the volunteers to provide informed consent was confirmed via a psychiatrist-conducted interview.

### Participants

A total of 22 patients with schizophrenia (mean age, 31.7±10.1 years; 12 males and 10 females) and 22 healthy controls (mean age 31.6±9.5 years, 12 males and 10 females) participated in this study (Table 1).

**Table 1:** Demographic and clinical characteristics of patients with schizophrenia and healthy controls.

	Schizophrenia (n = 22)	Control (n = 22)	p-value	Schizophrenia (n = 22)		
				Male (n = 12)	Female (n = 10)	p-value
Age (years)	31.7±10.1	31.6±9.5	p = 0.861^†^	29.7±9.6	34.1±10.7	p = 0.381^†^
Gender (male/female)	12/10	12/10	p = 1.000^‡^	12/0	0/10	p = 0.000^‡^
Handedness (% right)	100	100	p = 1.000^‡^	100	100	p = 1.000^‡^
Education (years)	13.6±2.5	14.9±2.4	p = 0.070^†^	14.0±1.7	12.7±2.7	p = 0.197^†^
Duration of illness (years)	9.2±6.6	-	-	7.3±6.4	11.3±6.7	p = 0.073^†^
Age at onset (years)	22.5±6.1	-	-	22.3±5.9	22.8±6.5	p = 0.843^†^
Clinical Global Impression	4.2±0.9	-	-	4.0±1.0	4.5±0.7	p = 0.343^†^
Positive and Negative Syndrome Scale						
Positive scale	18.4±5.2	-	-	17.5±4.5	19.3±5.9	p = 0.505^†^
Negative scale	21.1±6.0	-	-	20.1±4.7	22.1±7.1	p = 0.408^†^
General psychopathology scale	39.6±6.8	-	-	37.5±6.1	41.8±6.8	p = 0.197^†^

P-value was calculated by ^†^Mann Whitney U-test and ^‡^Chi-square test.

All patients were inpatients or outpatients of Chonbuk National University Hospital and were diagnosed with schizophrenia using the Diagnostic and Statistical Manual, Fourth Edition, Text Revised (DSM-IV-TR). A total of 22 patients with schizophrenia were recruited using the following criteria: schizophrenic diagnosis based on the Structured Clinical Interview for DSM-IV; no history of substance abuse/dependence over the last 6 months; no history of other neurological or psychiatric illnesses; and no diagnosis of anxiety or depression. The patients were assessed using the PANSS and the Clinical Global Impression (CGI). Four patients with schizophrenia received multiple antipsychotropic medications (amisulpride, n = 2; clozapine, n = 2; olanzapine, n = 2; paliperidone, n = 2; risperidone, n = 1); sixteen patients received a single antipsychotropic medication (amisulpride, n = 4; olanzapine, n = 1; paliperidone, n = 3; risperidone, n = 8); and two patients had not received antipsychotropic medication.

A total of 22 healthy controls were recruited from the following criteria: no schizophrenic symptoms based on the Structured Clinical Interview for the DSM-IV; no history of substance abuse/dependence over the last 6 months; and no history of neurological or psychiatric illnesses.

### MR imaging

The MR examinations were performed on a 3.0-T Magnetom Verio MR Scanner (Siemens Medical Solutions, Erlangen, Germany) with a 12-channel bird-cage head coil. The T1-weighted sagittal images were acquired using a three-dimensional magnetization-prepared rapid acquisition gradient echo (3D-MPRAGE) pulse sequence with the following parameters: repetition time (TR)/echo time (TE), 1900 ms/2.35 ms; field of view (FOV), 220×220 mm^2^; matrix, 256×256; slices, 176.

### Data post-processing and statistical analysis

MRI data were post-processed using Statistical Parametric Mapping software (SPM8, Wellcome Department of Cognitive Neurology, London, U.K.) with the DARTEL algorithm. Prior to data processing, all of the individual data were aligned with the anterior and posterior commissures line on the transverse plane. The MR images were processed using field bias correction to correct non-uniform fields and were then segmented to GM, WM, and cerebrospinal fluid (CSF) sections using tissue probability maps based on the International Consortium of Brain Mapping (ICBM) template for East Asian brains. The mean images of the individual GM and WM images were subsequently created. Individual GM and WM images were normalized to the Montreal Neurological Institute (MNI) template with a 1.5×1.5×1.5 mm^3^ voxel size and modulated for GM and WM volumes. Finally, all GM and WM images were smoothed with a 6-mm full-width at half-maximum isotropic Gaussian kernel.

For the group analysis, a two-sample t-test was performed to compare the GM and WM volumes over the whole-brain structures between patients with schizophrenia and healthy controls (p<0.05, FWE-corrected). For the analysis of the gender-dependent GM and WM volume alterations in schizophrenia, the total intracranial volume of each patient was used as a covariate in an analysis of covariation (ANCOVA, p<0.0001, uncorrected). The cluster size included more than 20 contiguous voxels. In this study, four regions of GM and three regions of WM showed decreased volumes in patients with schizophrenia. The correlations between either the PANSS scores or duration of illness and the GM volumes of the 4 ROIs or the WM volumes of the 3 ROIs were analyzed via multiple regression models. The GM and WM volumes as well as the MNI coordinates were analyzed using SPM8 and MRIcron software (www.mricro.com) [11, 22]. Spearman’s correlation test was performed using SPSS (version 20.0, IBM, Armonk, NY, USA).

## Results

### Demographic characteristics

No differences were observed between the patients with schizophrenia (n = 22) and healthy controls (n = 22) with respect to age, gender distribution, or length of education (Table 1). The mean CGI score of the patients was 4.2±0.9. The PANSS scores of the patients are based on a range of the means of 101 patients with schizophrenia in Kay’s study [23] concerning PANSS: positive scale, 18.4±5.2 (mean±SD); negative scale, 21.1±6.0; and general psychopathology scale, 39.6±6.8.

No sex differences were found regarding age, education, duration of illness, CGI, or PANSS scores between the male (n = 12) and female (n = 10) patients with schizophrenia (Table 1).

### Brain volume measurement

The GM volumes of patients with schizophrenia and healthy controls were 657.6±55.1 mL and 718.8±71.6 mL, respectively, whereas the WM volumes were 500.1±57.9 mL and 524.8±53.6 mL, respectively (Table 2). The GM volumes of patients with schizophrenia were significantly lower than those of the healthy controls (p = 0.012). The WM, CSF, and total intracranial volume of patients and healthy controls did not significantly differ.

**Table 2:** Differential intracranial component volumes in patients with schizophrenia and healthy controls.

Tissue	Schizophrenia (n = 22)	Healthy controls (n = 22)	p-value^1^	Schizophrenia (n = 22)		
				Male (n = 12)	Female (n = 10)	p-value
Gray matter	657.6±55.1 mL	718.8±71.6 mL	p = 0.012*	690.1±30.2 mL	618.6±53.7 mL	p = 0.007**
White matter	500.1±57.9 mL	524.8±53.6 mL	p = 0.074	526.5±62.3 mL	468.5±32.2 mL	p = 0.007**
Cerebrospinal fluid	433.1±86.3 mL	388.1±90.7 mL	p = 0.089	451.6±69.7 mL	411.0±102.1 mL	p = 0.323
Total intracranial volume	1590.9±153.7 mL	1631.7±155.9 mL	p = 0.534	1668.2±126.9 mL	1498.1±133.8 mL	p = 0.008**

P-value (*p<0.05**p<0.01) was calculated by Mann Whitney U-test.

Compared with male patients, female patients exhibited significantly decreased GM, WM, and total intracranial volumes (p = 0.007 for GM, p = 0.007 for WM, p = 0.008 for total volume; Table 2). However, the CSF volumes of the two groups did not significantly differ.

### Regional GM and WM volume changes in patients with schizophrenia

**Fig 1:**
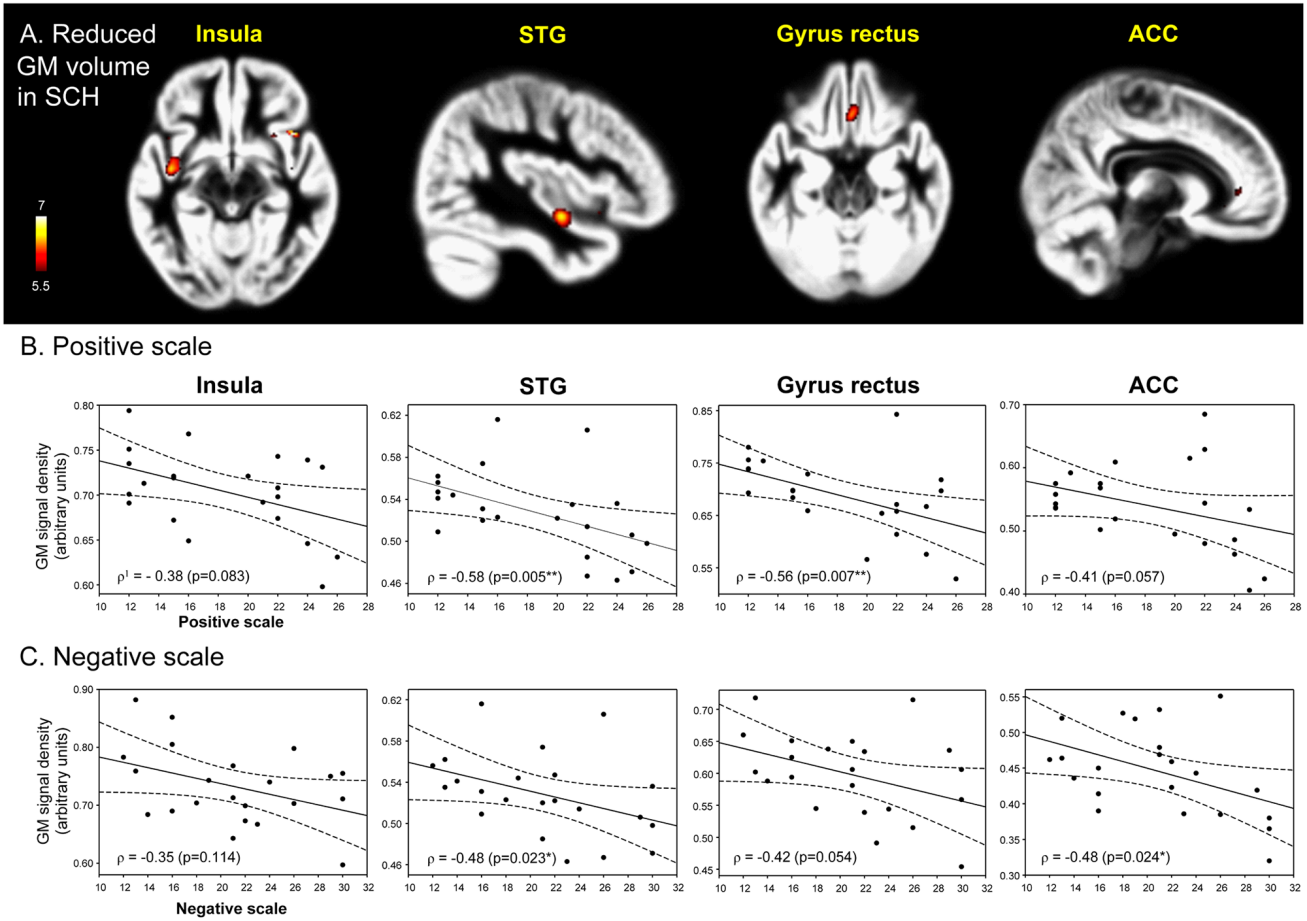
Decreased gray matter volumes and its correlation with clinical symptom severity in patients with schizophrenia compared with healthy controls. The patients with schizophrenia (n = 22) exhibited significantly lower GM volumes of the insula, STG, gyrus rectus, and ACC compared with the healthy controls (n = 22) (p<0.05, FWE) (A). The color-coded pixels were scaled to the range more than cut-off threshold (p<0.05). The GM volumes of the STG (p = 0.005) and gyrus rectus (p = 0.007) were negatively correlated with the positive scales (B) in the positive and Negative Syndrome Scale (PANSS) and those of the STG (p = 0.023) and ACC (p = 0.024) were negatively correlated with the negative scales (C). STG, superior temporal gyrus; ACC, anterior cingulate cortex. ^1^Spearman's rho. *p<0.05, **p<0.01, ***p<0.001.

Patients with schizophrenia exhibited significantly lower GM volumes in the insula, STG, gyrus rectus, and ACC compared with the healthy controls (p<0.05, FWE) (Table 3, Fig 1).

**Table 3:** Brain areas with significant gray matter volume alterations between patients with schizophrenia and healthy controls: two-sample t-test (p<0.05, FWE, cluster extent threshold: 20 voxels).

	t-value	MNI coordinates			Number of voxels	FWE- corrected p
		x	y	z		
**Healthy controls > Schizophrenia**						
Insula	7.22	42	18	-11	46	0.000***
Superior temporal gyrus	6.68	-41	-6	-12	165	0.002**
Gyrus rectus	6.23	2	29	-20	126	0.008**
Anterior cingulate gyrus	5.73	11	41	3	20	0.031*
**Schizophrenia > Healthy controls**						
None	-	-	-	-	-	-

P-value (*p<0.05, **p<0.01, ***p<0.001) was calculated by two-sample t-test

**Fig 2:**
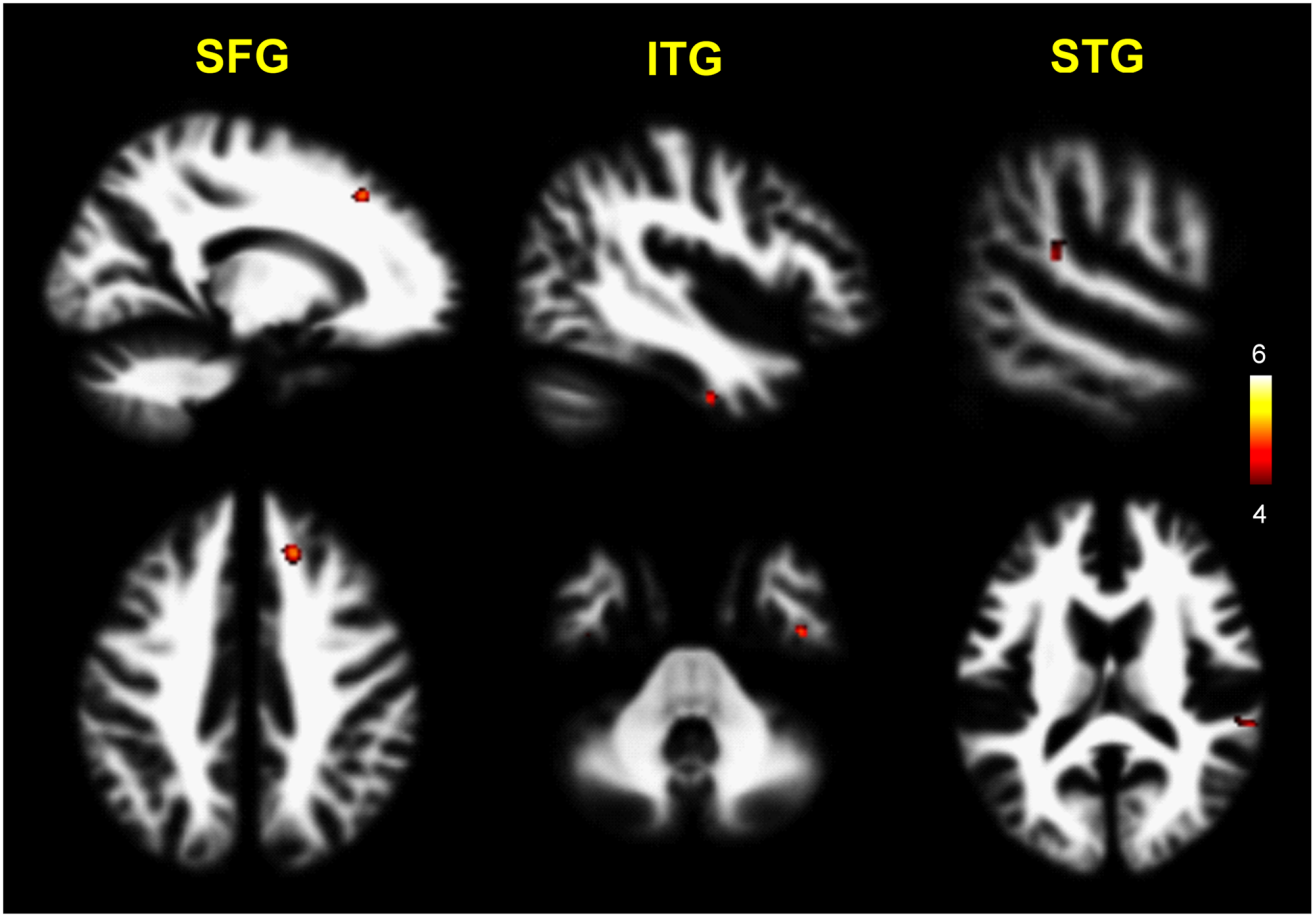
Decreased white matter volumes in patients with schizophrenia compared with healthy controls. The patients with schizophrenia (n = 22) exhibited significantly lower WM volumes of the SFG, ITG, and STG compared with the healthy controls (n = 22) (p<0.0001, uncorrected). The color-coded pixels were scaled to the range more than cut-off threshold (p<0.0001). SFG, superior frontal gyrus; ITG, inferior temporal gyrus; STG, superior temporal gyrus.

Also, the patients with schizophrenia exhibited significantly lower WM volumes of the superior frontal gyrus, inferior temporal gyrus, and STG compared with the healthy controls (p<0.0001, uncorrected) (Table 4, Fig 2).

**Table 4:** Brain areas with significant white matter volume alterations in patients with schizophrenia versus healthy controls: Two-sample t-test (p<0.0001, uncorrected, cluster extent threshold: 20 voxels).

	t-value	MNI coordinates			Number of voxels	Uncorrected p
		x	y	z		
**Healthy controls > Schizophrenia**						
Superior frontal gyrus	5.11	18	30	44	47	0.000***
Inferior temporal gyrus	4.85	44	-9	-38	21	0.000***
Superior temporal gyrus	4.62	62	-35	14	45	0.000***
**Schizophrenia > Healthy controls**						
None	-	-	-	-	-	-

P-value (***p<0.001) was calculated by two-sample t-test.

### Correlation of GM and WM volumes with PANSS scores

As shown in Fig 1, the positive scale scores of the PANSS were negatively correlated with the GM volumes of the STG (ρ = -0.58, p = 0.005) and gyrus rectus (ρ = -0.56, p = 0.007) in patients with schizophrenia, whereas the negative scale scores were negatively correlated with the GM volumes of the STG (ρ = -0.48, p = 0.023) and ACC (ρ = -0.48, p = 0.024).

Table 5 shows the correlations between the GM volumes and the positive/negative subscales of the PANSS. The GM volumes of the STG in patients with schizophrenia were negatively correlated with the positive scale scores of delusions, conceptual disorganization, grandiosity, and suspiciousness/persecution; those of the gyrus rectus were negatively correlated with the positive scale scores of delusions, conceptual disorganization, grandiosity, suspiciousness/persecution, and hostility; and those of the ACC were negatively correlated with the positive scale scores of conceptual disorganization and suspiciousness/persecution. The GM volumes of the STG were negatively correlated with the negative scales scores of blunted affect, poor rapport, difficulty in abstract thinking, and stereotyped thinking; those of the gyrus rectus were negatively correlated with the negative scales scores of stereotyped thinking; and those of the ACC were negatively correlated with the negative scales scores of emotional withdrawal and difficulty in abstract thinking.

**Table 5:** Correlations between GM volumes and positive/negative subscales in PANSS in patients with schizophrenia.

	Insula	STG	Gyrus rectus	ACC
**Positive scale**				
Delusions	ρ = -0.32 (p = 0.150)	-0.53 (0.011*)	-0.55 (0.009**)	-0.35 (0.109)
Conceptual disorganisation	-0.23 (0.298)	-0.53 (0.011*)	-0.50 (0.017*)	-0.44 (0.041*)
Hallucinatory behaviour	-0.30 (0.175)	-0.38 (0.081)	-0.32 (0.151)	-0.10 (0.662)
Excitement	-0.08 (0.730)	-0.31 (0.168)	-0.37 (0.089)	-0.22 (0.336)
Grandiosity	-0.39 (0.070)	-0.44 (0.042*)	-0.49 (0.021*)	-0.24 (0.289)
Suspiciousness/persecution	-0.33 (0.131)	-0.59 (0.004**)	-0.57 (0.007**)	-0.47 (0.029*)
Hostility	-0.24 (0.277)	-0.21 (0.347)	-0.50 (0.018*)	-0.10 (0.678)
**Negative scale**				
Blunted affect	-0.40 (0.068)	-0.49 (0.021*)	-0.36 (0.096)	-0.33 (0.131)
Emotional withdrawal	-0.27 (0.229)	-0.28 (0.212)	-0.37 (0.090)	-0.55 (0.008**)
Poor rapport	-0.14 (0.533)	-0.54 (0.009**)	-0.36 (0.104)	-0.39 (0.073)
Passive/apathetic social withdrawal	-0.26 (0.235)	-0.31 (0.163)	-0.30 (0.169)	-0.42 (0.052)
Difficulty in abstract thinking	-0.28 (0.207)	-0.48 (0.024*)	-0.23 (0.313)	-0.45 (0.036*)
Lack of spontaneity & flow of conversation	-0.33 (0.135)	-0.32 (0.154)	-0.35 (0.110)	-0.24 (0.291)
Stereotyped thinking	-0.21 (0.346)	-0.44 (0.042*)	-0.43 (0.049*)	-0.35 (0.108)

Data are presented as Spearman's rho (p-value). P-value (*p<0.05, **p<0.01) was calculated by Spearman’s correlation test. STG, superior temporal gyrus; ACC, anterior cingulate cortex.

No significant correlations were observed between the WM volumes and the positive/negative scales of the PANSS.

### Correlation between GM and WM volumes and the duration of illness

**Fig 3:**
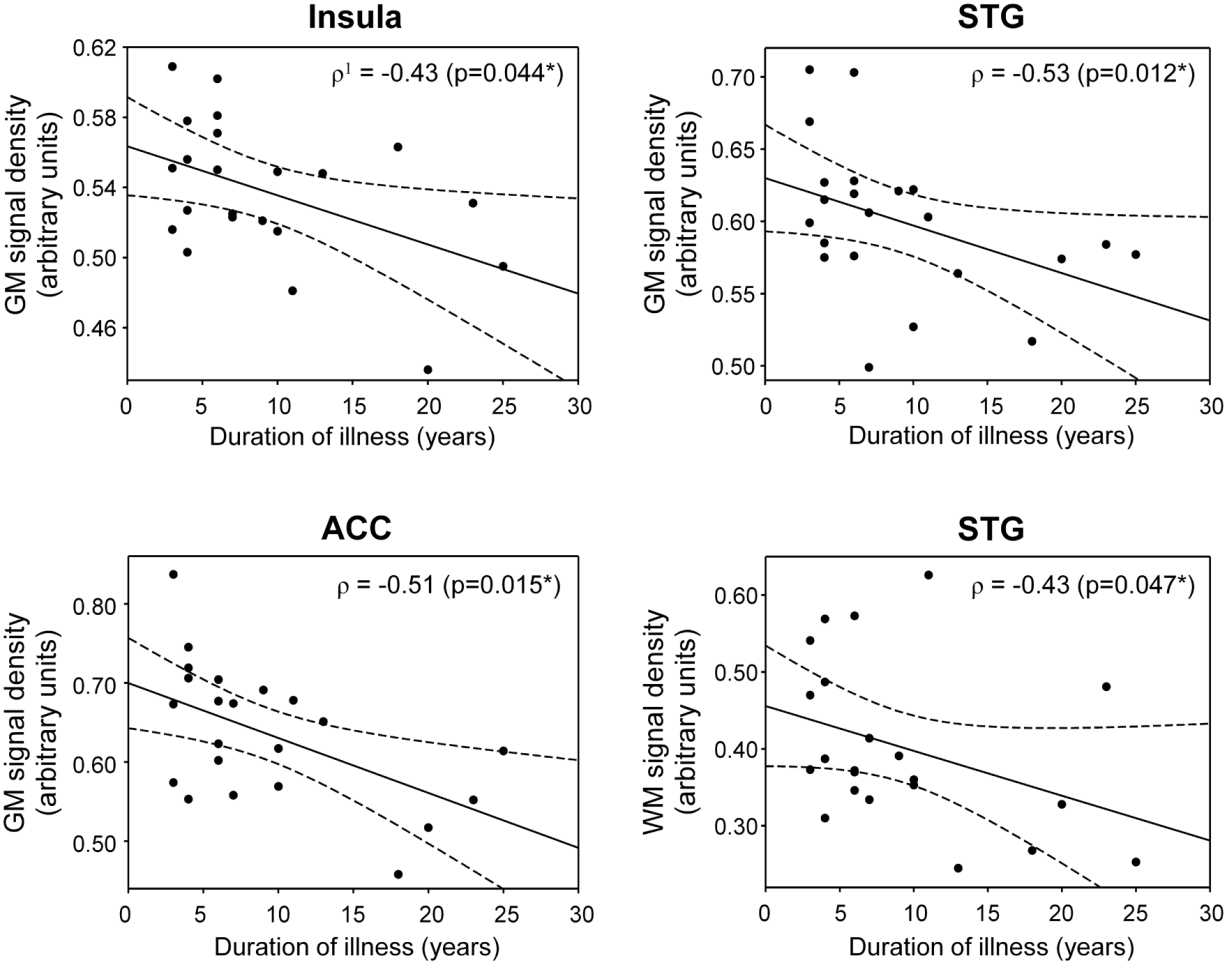
Correlations of the GM and WM volumes with the duration of illness in patients with schizophrenia. The GM volumes of the insula (ρ = -0.43, p = 0.044), STG (ρ = -0.53, p = 0.012), and ACC (ρ = -0.51, p = 0.015) were negatively correlated with the duration of illness in patients with schizophrenia. The WM volumes of the STG (ρ = -0.43, p = 0.047) were negatively correlated with the duration of illness in patients with schizophrenia. STG, superior temporal gyrus; ACC, anterior cingulate cortex. ^1^Spearman's rho. *p<0.05.

As shown in Fig 3, the GM volumes of the insula (ρ = -0.43, p = 0.044), STG (ρ = -0.53, p = 0.012), and ACC (ρ = -0.51, p = 0.015) were negatively correlated with the duration of illness in patients with schizophrenia (Fig 3). The WM volumes of the STG (ρ = -0.43, p = 0.047) were negatively correlated with the duration of illness in patients with schizophrenia (Fig 3).

### Gender differences in GM and WM volumes in patients with schizophrenia

**Fig 4:**
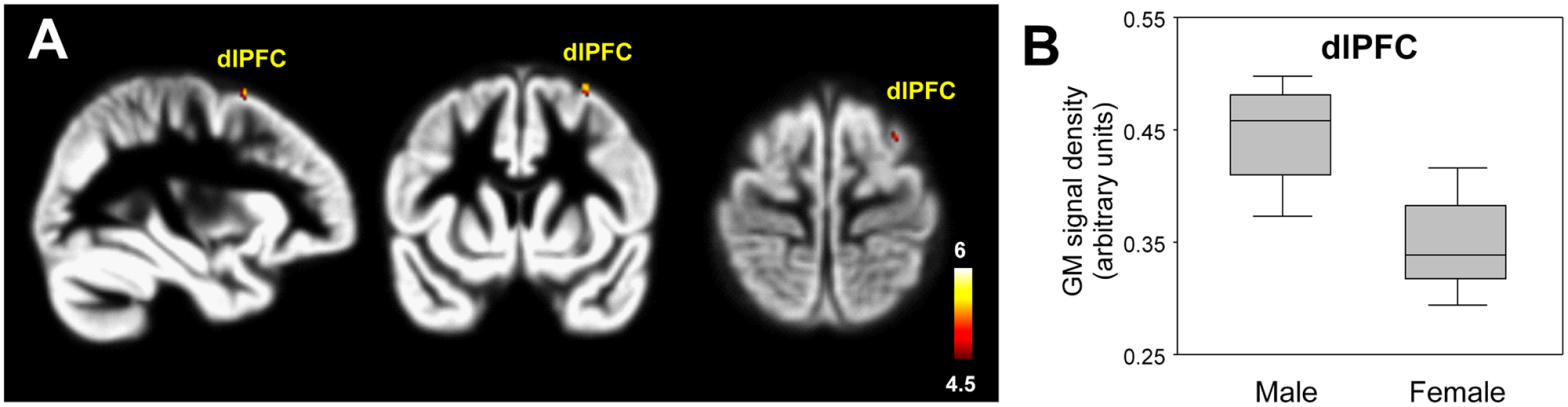
Gender difference in gray matter volumes in patients with schizophrenia. The female patients (n = 10) showed significantly lower GM volumes of the dlPFC compared with male patients (n = 12) (p<0.0001, uncorrected) (A). The color-coded pixels were scaled to the range more than cut-off threshold (p<0.0001). Box-plots show the medians, interquartile ranges, and the highest and lowest values of GM density of the dlPFC in male and female patients with schizophrenia (B). dlPFC, dorsolateral prefrontal cortex.

The gender comparison revealed that the female patients with schizophrenia showed significantly lower GM volumes in the dorsolateral prefrontal cortex (dlPFC) compared with male patients (p<0.0001, uncorrected) (Table 6, Fig 4). However, the WM volumes of the two groups were not significantly different.

**Table 6:** Brain areas with significant gray and white matter volume alterations in male vs. female patients with schizophrenia: ANCOVA (p<0.0001, uncorrected, cluster extent threshold: 20 voxels).

Brain areas	t-value	MNI coordinates			Number of voxels	Uncorrected p
		x	y	z		
**Gray matter**						
***Male > Female***						
dlPFC	5.57	32	11	65	29	0.000***
***Female > Male***						
None	-	-	-	-	-	-
**White matter**						
***Male > Female***						
None	-	-	-	-	-	-
***Female > Male***						
None	-	-	-	-	-	-

P-value (***p<0.001) was calculated by ANCOVA. dlPFC, dorsolateral prefrontal cortex.

## Discussion

In this study, MRI-based VBM with DARTEL was used to evaluate the GM volume alterations in patients with schizophrenia and their correlations with PANSS scores. Total GM volumes were significantly lower in patients with schizophrenia than in healthy controls, particularly in the insula, STG, gyrus rectus, and ACC (p<0.05, FWE). In particular, the GM volumes of the STG and gyrus rectus were negatively correlated with the positive scales of the PANSS, whereas those of the STG and ACC were negatively correlated with the negative scales.

A GM volume abnormality of the STG is the most frequently reported finding in patients with schizophrenia [14,24,25]. We also observed a significant decrease in the GM volume of the STG in patients with schizophrenia in the present study. The STG plays an important role in the production, interpretation and self-monitoring of language [26]. GM volume abnormalities in the STG have been linked to symptom severity in patients with schizophrenia. Egashira et al. [17] reported that patients with late- and early-onset schizophrenia exhibit decreased GM volume in the STG compared with healthy controls. The STG has also been implicated in the experience of auditory hallucinations in patients with schizophrenia [26,27]. Barta et al. [28] reported that GM abnormalities in the STG are strongly correlated with the severity of auditory hallucinations. Menon et al. [29] suggested that GM abnormalities of the STG are associated with positive symptoms in schizophrenia. Conversely, Kim et al. [30] suggested that abnormalities in the STG are associated with negative symptoms. In our study, the GM volumes in the STG were negatively correlated with both the positive and negative scale scores on the PANSS. The duration of illness in patients with schizophrenia was also negatively correlated with the GM volumes in the STG. GM volume decreases in the STG are likely related to psychosis severity, disorganized dimensions, and formal thought disorder in patients with schizophrenia [31–33].

One interesting finding of this study is the decreased GM volume of the insula in patients with schizophrenia, which is consistent with the GM volume abnormalities in the insula in schizophrenia observed in previous studies [17,34,35]. Postmortem studies [36,37] have revealed that the reduction of insula volume stems from decreases in the neuronal and glial somal sizes in layer II and III due to poor development and heterogeneity in the upper layers of the insula. Many similar studies [38–40] have reported abnormalities in cortical thickness and protein expression in the insula related to schizophrenia. At Risk Mental State (ARMS) participants who subsequently develop psychosis exhibit decreased GM volumes in the insula compared with those who do not become psychotic [41]. In addition, the GM volumes of the insula were negatively correlated with the duration of illness in the present study. These results indicate that GM volume abnormalities in the insula are closely associated with the psychopathology of schizophrenia.

Decreased GM volumes in the ACC are associated with decreased inhibitory interneurons and projection neuron loss in patients with schizophrenia [42–44]. The ACC is divided into two areas: the dorsal cognitive division is part of a distributed attentional network, whereas the ventral affective division is primarily involved in assessing the salience of emotional and motivational information as well as the regulation of emotional responses [45]. In our study, patients with schizophrenia exhibited decreased GM volumes in the dorsal ACC. An fMRI study [46] revealed that patients with schizophrenia exhibit decreased BOLD signals in the dorsal portion of the ACC during working memory tasks with attention and inhibitory processes. Taken together, the results of the aforementioned fMRI and VBM studies suggest that the dorsal ACC is associated with perceptual disturbances in patients with schizophrenia. Several studies [47–49] have reported that GM volume abnormalities in the ACC are associated with the positive/negative symptoms of schizophrenia. In our study, the GM volumes of the ACC were negatively correlated with the negative scales.

In contrast to a previous DARTEL-based VBM study [17], we observed decreased GM volumes in the gyrus rectus in patients with schizophrenia. Frontal lobe dysfunction is related to cognitive and affective deficits in patients with schizophrenia [46]. The gyrus rectus, which is an extension of the anterior cingulate onto the frontal lobe, has been implicated in schizophrenia. In addition, the GM volumes of the gyrus were negatively correlated with both the positive and negative scales. We assume that the decreased GM volumes in the gyrus rectus play an important role in the psychopathology of patients with schizophrenia. Supporting this finding, Wilke et al. [50] observed decreased GM volumes in the gyrus rectus of patients with schizophrenia compared with healthy controls.

We also found some evidences of decreased WM volumes in the superior frontal gyrus and superior/inferior temporal gyri in patients with schizophrenia. It is now well known that the cognitive and affective deficits associated with schizophrenia are correlated with frontal lobe dysfunction [46]. The superior frontal gyrus is responsible for emotionally and instinctively organized aspects of behavior [51]. When this area is damaged, impairments emerge in social behaviors such as planning, judgment, and decision making. Morphometric and diffusion tensor imaging (DTI) studies [52,53] have reported that patients with schizophrenia show WM loss in the frontal and temporal lobes. According to another DTI study [54], patients with schizophrenia showed decreased anisotropy in the WM of the STG. One interesting finding in our study is that decreased GM and WM volumes in the STG in schizophrenia were respectively correlated with duration of schizophrenia. It is assumed that the GM and WM volume abnormalities of the STG are linked to the pathology of schizophrenia.

More importantly, our study revealed sex-related GM volumetric differences in patients with schizophrenia. Female patients showed significantly decreased GM volumes in the dlPFC compared with male patients. The dlPFC is a part of the frontal-subcortical neural circuitry that modulates mood and emotional processing [1,55]. A previous morphometric study [56] found that female patients with schizophrenia show structural abnormalities in the frontal lobe compared with male patients. Another study [57] demonstrated reduced orbital prefrontal GM volume in female patients, which was associated with poorer premorbid functioning, more severe negative symptoms, and depression. In addition, a functional connectivity study [58] demonstrated that female patients with schizophrenia show a reduction in global functional connectivity compared with male patients. These findings support the view that the decreased GM volume of the dlPFC is associated with sex differences in clinical and etiological factors or cognitive functions. GM volumetric abnormalities of the dlPFC are considered as a potential morphometric biomarker for discriminating between male and female patients with schizophrenia.

Our current study is subject to some limitations. First, the population of participants was too small to increase the statistical power. Second, this study dealt with chronic patients with schizophrenia, and thus a longitudinal study involving first-episode and chronic patients is needed to gain more valuable information on the morphological abnormalities of the GM and WM in patients with schizophrenia. Third, the possibility that antipsychotic medications might affect GM and WM volume variations in patients with schizophrenia was not considered. Fourth, we did not apply the Assessment of Positive Symptoms (SAPS) or the Assessment of Negative Symptoms (SANS) scales regarding schizophrenia symptom severity.

## Conclusions

This study revealed the GM and WM volumetric differences between healthy controls and patients with schizophrenia using DARTEI-based VBM as well as the correlations between the localized GM and WM volume alterations and PANSS scores. Our findings suggest that the GM and WM volume abnormalities of the STG are associated with the psychopathology of schizophrenia. These findings might be helpful in assessing the malfunctional connectivity associated with schizophrenic symptoms.

WJ.
